# Laser fluorescence assessment of dental caries arrest with two silver fluoride agents in patients with special needs– a preliminary report

**DOI:** 10.1007/s10103-024-04038-7

**Published:** 2024-04-01

**Authors:** Lydia See, Sobia Zafar, David Fu, Diep H. Ha, Laurence J. Walsh, Claudia Lopez Silva

**Affiliations:** 1 The University of Queensland, School of Dentistry, 288 Herston Road, Herston , Brisbane, QLD, 4006 Australia; 2 Oral Health Services, Metro North Oral Health Center, Queensland Health, Brisbane, Australia

**Keywords:** Dental caries, Silver fluoride, DIAGNOdent, Fluorescence, Special needs, Caries arrest

## Abstract

**Purpose:**

While silver diamine fluoride has been used extensively for caries arrest and desensitising, silver fluoride (AgF) at neutral pH may also have value as a minimally invasive dental caries treatment. This study explored the effectiveness of two AgF products (AgF/KI and AgF/SnF_2_) when used in adult patients with special needs (SN) who had high caries risk and salivary gland hypofunction.

**Methods:**

This split-mouth clinical study, over two appointments 3-months apart, compared the impact of a single application of AgF/KI (Riva Star Aqua, SDI) and AgF/SnF_2_ (Creighton Dental CSDS, Whiteley) on matched carious lesions in the same arch, by clinical visual-tactile (cVT) assessment of caries status and laser fluorescence (LF, DIAGNOdent) evaluation of bacterial load in the lesions, using repeated measures analysis.

**Results:**

Twelve participants were recruited in the study. A total of 56 teeth (28 pairs) were included. Both AgF products gave a significant decrease in caries activity as measured by cVT (*P* < 0.0001) and LF (*P* = 0.0027). There were no statistically significant differences between the two AgF treatments, with response rates for improvements in active lesions of 92% in the AgF/KI arm, and 96% in the AgF/SnF_2_ arm. There was no effect of tooth type, lesion type, arch type, plaque metabolism and plaque area at the site level on outcomes, nor was there a clustering effect of sites in a patient level analysis. Overall, LF was superior to cVT for detecting lesions that still progressed despite treatment (*P* = 0.0027).

**Conclusion:**

A single application of AgF/KI or AgF/SnF_2_ has high predictability (over 90%) for achieving arrest in active caries lesions in adult patients with SN and high caries risk. Clinical assessment should use visual-tactile examination combined with LF readings to detect lesions that are still progressing and that require additional treatments. Future studies should compare these AgF modalities with SDF and explore factors such as time between applications and the need for repeated applications.

**Trial registration:**

The study was registered with the Australian Clinical Trials Registry (ACTRN12621001139864p) on 23/08/2021.

## Introduction

There is a great need for effective methods for arresting dental caries in patients with special needs, since they have poorer oral health and less access to timely dental care compared to others in the community [1]. Individuals with special needs often present for clinical treatment with high rates of untreated dental caries [2], and high levels of dental plaque [3]. Hence, methods that can be used to arrest carious lesions and stabilise them would be of great value. While there has been considerable recent interest in silver diamine fluoride (SDF) for caries arrest in children and in older adults [4], with high rates reported for caries arrest [5–7], this treatment modality can cause transient soft tissue irritation because of its alkaline pH [8], which comes from its ammonia ingredient. As a result, there has been renewed interest in silver fluoride formulations that lack ammonia. These may be better suited to situations involving children and patients with special needs whose ability to tolerate dental treatment is limited, and where discomfort from soft tissue irritation would be above the threshold of tolerance for a dental intervention.

There is a strong evidence base supporting the use of SDF for caries arrest in primary teeth in children, and in permanent teeth in older adults, including those living in residential aged care facilities [5–7, 9, 10]. While the application technique for SDF is simple and quick, staining of treated tooth structure occurs, which is a concern when treated lesions are in the aesthetic zone and the discolouration will be visible to others [11, 12]. This has led to the development of SDF formulations such as Riva Star™ (SDI, Bayswater, VIC, Australia) that are followed by the immediate application of potassium iodide (KI), which is intended to precipitate out excess silver ions, and slow down or prevent the development of staining [8].

As well as SDF/KI, there are now also novel formulations of silver fluoride (AgF) available, including where 38% AgF is applied and then followed by KI as a stain reducing agent (Riva Star Aqua™, SDI, Bayswater, VIC, Australia) (AgF/KI). A further variation is where the 40% AgF is applied, and immediately followed by 10% stannous fluoride (SnF_2_) (AgF/SnF_2_), such as with Creighton Dental Caries Status Disclosing Solution (CSDS™, RM Creighton Dental Pty Ltd - Dental caries detecting stain, manufactured by Whiteley Medical, Newcastle, NSW, Australia). To date, there has been limited research into the use of either of these recently developed AgF/KI or AgF/SnF_2_ treatments for dental caries arrest, including in adults with special needs. Positive results have been reported from studies conducted in Sydney, Australia for CSDS used in elderly patients with cognitive disability whose cooperation was limited [8, 13]. In these reports, caries arrest outcomes were assessed using clinical visual-tactile scores of lesion status [8, 13, 14].

Based on this background, the present study was designed to explore the effectiveness of AgF/KI versus AgF/SnF_2_ when used in ambulant adult patients with special needs attending a community-based special needs dentistry clinical service. The caries status was scored clinically and, in addition, the bacterial load of the dentine in the cavity floor was tracked in a quantitative manner using laser-induced fluorescence with the DIAGNOdent™. This device measures fluorescence generated by fluorophores in bacteria, including protoporphyrin IX, within tooth structure, including within the dentine of the cavity floor, as in the present study. A semiconductor diode laser emits pulses of 655 nm light [15], which elicits fluorescence in the near infrared region (longer than 700 nm). The laser emissions and photodiode sensor response are both gated, to reduce the impact of ambient light [15]. The fluorescence readings range from 0 to 99 [16].

In the present study, the DIAGNOdent was used to compare the load of bacteria in carious lesions at baseline and at 3 months after a single treatment with AgF/KI or AgF/SnF_2_. Past studies have used DIAGNOdent readings longitudinal monitoring of coronal and root surface caries lesions, and for assessing the outcome of preventive interventions, including in studies of protocols for arresting root surface caries in patients with special needs [17]. The DIAGNOdent has a high reproducibility and reliability for dental caries detection, with reported values for sensitivity and specificity of 0.81–0.83 and 0.78–0.95 for root surface caries [18], and 0.88 and 0.84 for caries on the facial or lingual smooth surfaces of teeth [19]. In the present study, both DIAGNOdent scores and traditional clinical visual-tactile (cVT) assessments (clinical scores) of carious lesions were used to track outcomes from the two forms of AgF therapy, once all dental plaque had been removed from the teeth and from any cavities.

## Materials and methods

### Study design

A split mouth study design was used to compare the clinical outcomes and bacterial load for AgF/KI (Riva Star Aqua, SDI) and AgF/SnF_2_ (Creighton Dental CSDS, Whiteley). The split mouth design with comparable lesions on opposite sides of the arch was chosen to provide the most valid comparison of products used within the one patient [20, 21]. It allowed for elimination of the inter-subject variability from the intervention being studied and a lower sample size [20, 21]. As the two silver fluoride materials were applied topically in small volumes and the solutions were localised to the site of application, no cross-contamination effects were expected [22]. Both clinical and DIAGNOdent assessments were made once all plaque had been removed from the surface of the cavity or the tooth. A power analysis (Statmate™ 2 for Windows version 2.0, GraphPad Software, San Diego, USA) was used to estimate the required sample size for the study. A change in the fluorescence reading of more than 3 was considered clinically important. For laser fluorescence scores with an expected standard deviation (SD) of each group of 15 and an expected correlation (r) among pairs of 0.95, a sample size of 12 would detect a difference between means of 5.18 at a power of 95% and a significance level of 0.05 with a paired two-tailed test.

### Subjects

Patients who were 18 years and older with special needs (i.e., intellectual disability, medical, physical, or psychiatric conditions) attending a special needs dentistry public clinical service were eligible for recruitment. All subjects recruited into the study had at least one pair of similar carious lesions (cavitated or non-cavitated, located either on the root, coronal or mixture) on smooth surfaces (easily accessible – buccal, or interproximal) of the same type of teeth in the same arch and on opposing sides. The selected teeth were either asymptomatic or had mild and reversible pulpal symptoms (non-lingering pain to cold, hot, and air) and no periapical pathology (no clinical signs of tenderness to percussion or chewing, no draining sinuses or swellings; no radiographic signs of radiolucency or widened space around the tooth apex). Participants were required to be able to attend two appointments and be sufficiently compliant to allow the application process of the two silver fluoride materials to occur.

Participants who had known allergies or adverse reactions to any components of the materials or who were uncooperative/non-compliant were excluded. Likewise, any teeth where carious lesions were associated with clinical and radiographic signs and symptoms of irreversible pulpal and periapical inflammation (prolonged unstimulated toothache, pain lingering for longer than a few minutes after cold/hot stimuli, or tenderness to chewing/functioning) were excluded.

The study was registered with the Australian Clinical Trials Registry (ACTRN12621001139864p). Ethics approval for the study was granted by Metro North Hospital and Health Services (HREC/2021/QRBW/73,736) and by the University of Queensland (2021/HE002354).

### Clinical procedures

Participants were blinded to the material used and to the sites which were selected. To eliminate bias, the following were undertaken. Firstly, an online random number generator (https://www.calculator.net/random-number-generator.html) was used to allocate the treatment site within the lesion pairs (right versus left), with AgF/KI being used on the right for an even number, and on the left for an odd number. Secondly, the review appointment at 3 months was undertaken without reference to clinical records of the initial treatment appointment. Thirdly, data collected were deidentified and coded prior to statistical analysis.

Subjects were treated by two clinicians (a specialist in special needs dentistry, and a specialist in training). The clinical protocol is shown in Table 1. Each of the sites was photographed, then the overlying dental plaque was stained using a multicolour dental plaque diagnostic tool (GC Tri-Plaque ID™ gel, GC Corporation, Tokyo, Japan), in line with the manufacturer’s instructions, to identify plaque age, thickness, and microbial fermentation. This material contains multiple pH responsive dyes and includes sucrose as a substrate for acid production. Young dental plaque (less than 24 h old) stains pink, mature plaque (over 24 h old) stains dark blue/purple, and mature plaque that is producing acid from microbial fermentation stains light blue [23, 24]. Plaque deposits were stained at initial and review appointments, and then photographed. Images were then scored for the percent pixel area of the plaque (calculated using Adobe Photoshop™ version 22.4.3) using previously published methods [23, 24]. All plaque was then removed from the teeth, including from within the cavities to be scored, by a dental prophylaxis with a rubber cup using a non-fluorescent prophylaxis paste (Enamel Pro® Prophy Paste, Premier Dental Co., Plymouth Meeting, United States of America). After the teeth had been dried with compressed air, the lesions were photographed again, then examined and probed to score their clinical status. The clinical caries activity (clinical scoring) was scored as being active or inactive [18, 25], using the criteria shown in Table 2 [26].

**Table 1:** Clinical protocol

**Baseline appointment** 1. Confirmation of tooth sites 2. Allocation of sites using randomisation technique 3. Photographs #1 of sites 4. GC TriPlaque ID gel staining 5. Photographs #2 of sites (for plaque analysis) 6. Prophylaxis 7. Clinical scoring of lesions 8. Recording DIAGNOdent scores of the lesions 9. Photographs #3 of sites 10. AgF application as per manufacturer’s protocol 11. Photographs #4 of sites 12. Oral hygiene and dietary advice
**Review appointment** 1. Repeat all steps 3–9 above to assess outcomes 2. Oral hygiene and dietary advice

**Table 2:** Clinical scoring of carious lesions [26]

Variable	Definition
**Active lesion**	Visual: whitish/yellowish opaque with loss of lustre for non-cavitated surfaces; cavity visible to naked eye for cavitation Tactile: rough when tip of probe passes over surface for non-cavitated surfaces; soft/leathery on gentle probing for cavitated surfaces
**Inactive lesion**	Visual: whitish, brownish, or black, shiny for non-cavitated surfaces; shiny on cavitated surfaces Tactile: hard and smooth for non-cavitated surfaces when probe tip is moved gently across surface; hard on probing with gentle pressure on cavitated surfaces.

To quantify the load of bacteria, present in the carious dentine before and after treatment, laser induced fluorescence readings were taken using the DIAGNOdent Classic (model 2095, KaVo, Biberach, Germany) fitted with a wide area scanning tip (“B tip”), with an area of 1 mm^2^. The DIAGNOdent device was calibrated according to the manufacturer’s instructions using a ceramic disc prior to each session of use. DIAGNOdent readings were made for each cavity, and the peak value was agreed by both clinicians.

The paired lesions were then treated with either AgF followed by KI, or by AgF followed by SnF_2_, following the manufacturer’s instructions. A timer was used to ensure a one-minute application time for AgF. No gingival protection or cavity protection were applied. The treated sites were photographed, and the patients then dismissed after providing tailored oral hygiene and diet instructions.

At the 3-month recall appointment, the same clinical protocol for scoring the lesions was followed, involving the same steps of initial photography, multicolour plaque disclosing, and dental prophylaxis prior to visual and tactile examination and assessment using the DIAGNOdent.

### Data analysis

Clinical data were de-identified prior to data entry. Statistical analysis was undertaken using Jamovi software version 2.2.5 (The Jamovi project, Sydney, Australia). A P value of < 0.05 was used as the threshold for statistical significance. Prior to statistical analysis, all data sets were tested for normal distribution using the Shapiro-Wilk normality test. Based on Levene’s homogeneity of variance test, all data sets had comparable variance. Where data sets had normal distributions, the independent and paired-sample Student T-test were used to compare the means of continuous data. Any situations where data sets did not follow a normal distribution were dealt with by using the Wilcoxon rank or Mann-Whitney U tests. For categorical data, the Chi-square test of independent association and the McNamar test were used.

The outcomes assessed were changes in the clinical activity score of the lesion, and changes in DIAGNOdent readings, from baseline to review. To categorise responses, changes in the fluorescence reading of greater than 3 were considered relevant, in line with instructions from the manufacturer. Lesions where changes were 3 or less were considered to be stable. An increase in reading by more than 3 indicated lesion progression, and a drop in reading by more than 3 indicated improvement. As well as analysis at the site level for the influence of various factors, analysis was also done of responses at the patient level (i.e., analysis of uniformity), to assess the potential influence of patient-related factors on the clinical effectiveness of the two different AgF products.

## Results

### Participants

There were 12 participants (7 M/5F). These ranged in age from 34 to 73 years (mean 50.4 years, median 47.5 years). In terms of their profile of special needs, 8 had psychiatric conditions, 3 were medically complex, and one patient was a combination of both. All twelve participants were taking at least one medication with known hyposalivatory actions and had signs of hyposalivation (recorded as the Challacombe score). Polypharmacy was common, with 8 of the 12 participants taking more than 5 medications.

All 12 participants completed the required two appointments. The actual interval between the baseline and the 3-month review appointments varied from 70 to 140 days (mean 94.2 days, median 91.0 days). Within the 12 participants there were 28 treated pairs. Of the 56 teeth, 12 were in the maxillary arch and 44 in the mandibular arch, with 48 teeth having cavitated carious lesions into the dentine and 8 teeth having incipient root surface caries. Caries locations on the smooth surfaces were a mixture of root caries and cervical third caries. All lesion pairs had lesions of the same type, and all included lesions were readily accessible. By tooth type, there were 8 molars, 19 anterior teeth and 29 premolar teeth.

### Clinical scores

**Fig. 1:**
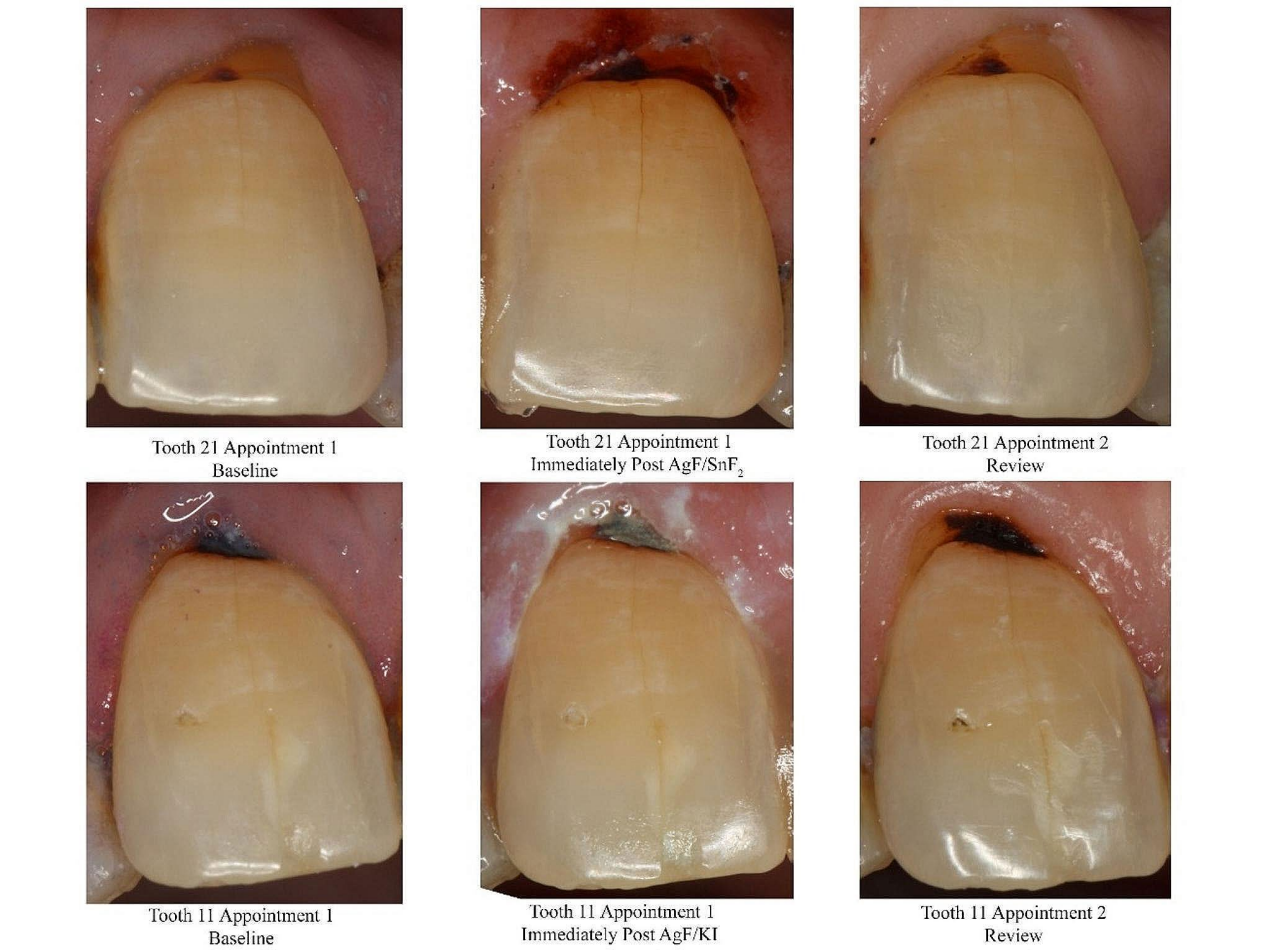
A sample of serial photographs for each tooth, showing the situation at baseline, immediately after AgF application, and at the later review. There was immediate darkening seen for the lesion treated with AgF/SnF_2_. Both types of AgF caused darkened lesions at the later review appointment

Overall, there were no statistically significant differences between the two treatment groups at baseline, where 49 of the 56 teeth had active lesions (25 in the AgF/KI arm and 24 in the AgF/SnF_2_ arm), and 7 were inactive (3 in the AgF/KI arm and 4 in the AgF/SnF_2_ arm). Figure 1 demonstrates images taken before, and immediately after AgF application, and at review.

There was a marked improvement in the activity profile of sites for each of the two AgF treatments (*P* < 0.0001 for both treatment arms, using the McNemar Test). At 3-month review, there were 53 teeth that were clinically assessed as now being inactive (26 in the AgF/KI arm and 27 in the AgF/SnF_2_ arm), and 3 teeth were assessed as being still active (2 in the AgF/KI arm and 1 in the AgF/SnF_2_ arm). This gave response rates for improvements in active lesions as follows: 23/25 or 92% in the AgF/KI arm, and 23/24 or 96% in the AgF/SnF_2_ arm. There were no statistically significant differences between the two treatment groups (*P* = 0.788).

Analysis of response patterns for clinical scores showed no influence of tooth type (anterior/premolar/molar, *P* = 0.475), lesion type (cavitated/non-cavitated, *P* = 0.061), or arch (maxillary/mandibular, *P* = 0.597).

### DIAGNOdent readings

**Fig. 2:**
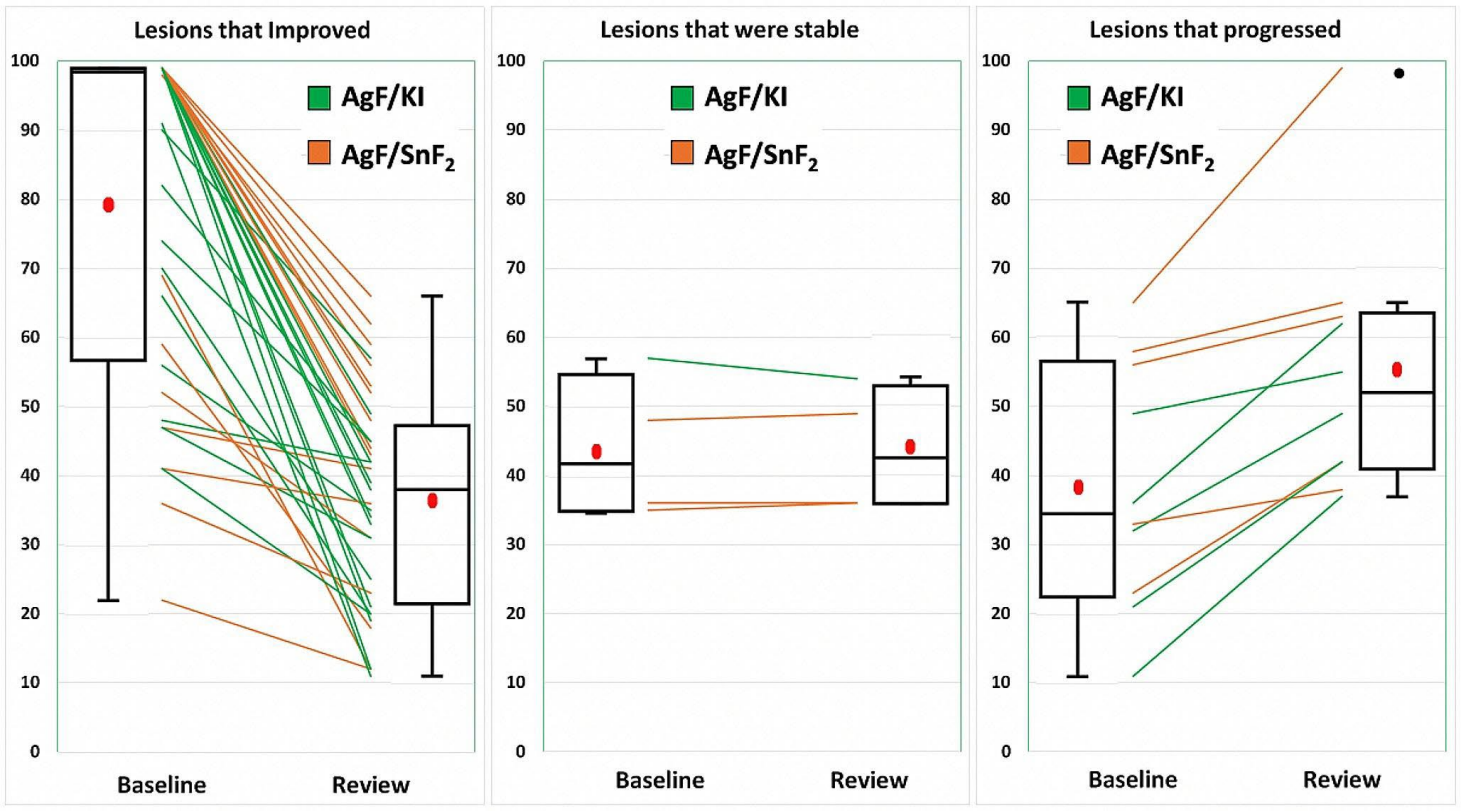
Composite panel showing the three response patterns for the two different AgF products used in the study. Response patterns were classified according to the extent of change on DIAGNOdent scores (> 3). Samples where the baseline score and the review score were both 99 are not included in this analysis (*N* = 6). Left panel shows lesions that improved (scores reduced between baseline and 3 months, *N* = 36); Middle panel shows lesions that were stable (*N* = 4); Right panel shows lesions that progressed (*N* = 10). The green coloured lines are AgF/KI while orange coloured lines are AgF/SnF_2_, showing fluorescence readings for individual lesions at baseline and at review. In the box and whisker plots, the box is the interquartile range, the median is shown as a horizontal line, and whiskers shows the maximum and minimum. The arithmetic mean is shown as a red ellipse

DIAGNOdent data is presented in Fig. 2. Mean DIAGNOdent readings for the 56 sites at baseline were similar across both groups AgF/KI 73.5 (95% CI 63.0-84.1), and AgF/SnF_2_ 70.2 (95% CI 59.4–81.0), with a mean difference of 3.3, which was not significantly different (*P* = 0.726). At 3 months, these readings reduced in both groups AgF/KI 41.2 (95% CI 33.4–49.0) and AgF/SnF_2_ 52.9 (95% CI 43.1–62.6). With both treatments, the reduction between baseline and at three months was significant with each treatment (*P* < 0.0001 and *P* = 0.0039, respectively), but there was no significant difference in mean reduction score between the two types of materials (*P* = 0.429). The mean difference between the two treated groups at review rose to 11.7, but this did not reach the threshold for statistical significance (*P* = 0.066).

In terms of response patterns, based on the threshold change of 3 in the DIAGNOdent reading, with AgF/KI, 20 sites improved, 7 progressed, and 1 was stable with no change. With AgF/SnF_2_, 16 sites improved, 9 progressed, and 3 were stable (Fig. 2). Overall, the DIAGNOdent was superior to clinical examination for detecting situations where lesions had progressed over the 3-month interval (*P* = 0.0027). In terms of sites, just over 28% showed progression based on the change in DIAGNOdent readings, while only just over 5% of sites showed progression according to clinical scoring. Analysis of response patterns for DIAGNOdent readings showed no influence of tooth type (*P* = 0.759), lesion type (*P* = 0.628), or arch (*P* = 0.946). At the patient level, there were no significant influences in response patterns to AgF/KI and AgF/SnF_2_ across participants in an analysis of uniformity by outcome.

### Plaque metabolism and plaque area

There were no statistically significant differences in plaque characteristics between baseline and review (based on the percentage distributions for pink (*P* = 0.6042, dark blue/purple (*P* = 0.7657), and light blue-coloured areas of plaque (*P* = 0.7860), and likewise in the total plaque area from baseline to review (*P* = 0.3491).

## Discussion

The results of this clinical study demonstrate the clinical efficacy of AgF/KI and AgF/SnF_2_ in arresting carious lesions in patients with special needs, using the accepted method of clinically scoring carious lesion activity, and using laser-induced-fluorescence. These findings for recently developed AgF treatment approaches add considerably to what is known for both AgF and for SDF in the literature [5–7].

Overall, both AgF/KI and AgF/SnF_2_ were highly effective at arresting active lesions, with no statistically significant differences between them. Future studies should compare AgF/KI and AgF/SnF_2_ with SDF and with SDF/KI, and to AgF alone, as well as consider the influence of factors such as the application time used for various products, since these may range from as short as 10 s to as long as 3 min [27]. An application time that is long will be problematic for uncooperative patients, including children, and adults with special needs [11, 14]. Likewise, future studies need to consider whether multiple applications are effective for arresting lesions that continue to progress after a single treatment, and to explore the influence of variables such as lifestyle and the use of home oral care products on outcomes from silver fluoride treatments of various types.

The split mouth approach showed that factors such as tooth type, lesion location, arch type and plaque composition were not significant factors in determining the outcome. Moreover, as the subjects in this study all were using medicines with hyposalivatory actions, it is noteworthy that high rates of arrest for active lesions were achieved (above 90%), despite impaired salivary function, which itself is a major risk factor for caries progression.

In any situation where tracking a lesion over time is important, confidence in the detection ability of the assessment method being used is paramount. The present results indicate that using traditional visual examination and probing to assess stability versus improvement or progression is not as sensitive as following changes in fluorescence scores. This is not unexpected given that clinical assessment can only detect active vs. inactive lesion and does not assess for depth of penetration of bacteria. Moreover, the DIAGNOdent provides a quantitative and objective means for assessing carious lesions, while probing lesions to assess their hardness is qualitative and more variable nature because of factors such as probing force and probe shape.

Important caveats that arise because of the high sensitivity of the DIAGNOdent are that (1) all traces of dental plaque must be removed from the tooth surface or cavity that is being assessed, (2) the probe tip is kept clear of any oral fluids or debris, and (3) a non-fluorescing prophylaxis paste used to clean the tooth surface, to prevent false positive results. When these three key requirements are met, very strong performance has been shown in past clinical studies for tracking enamel lesions on smooth surfaces over time [28]. Hence, the present study supports the view that cavitated lesions undergoing a caries arrest protocol, in teeth with symptoms of reversible pulpal pathology, should be monitored over time using laser fluorescence.

When such monitoring is being done, it is essential to consider the use of a threshold for meaningful change in readings (such as 3) and the issue of what happens when the baseline score is out of scale (i.e., above 99, which is the maximum shown on the display). A lesion with very high levels of bacteria may take some time to show a reduction in score, although such changes have been seen in 12-month studies [17]. Adding to this, published recommendations on DIAGNOdent reading cut-offs for the extent of bacterial involvement vary [18, 19, 29, 30]. For lesions on smooth surfaces, a cut-off reading of 8 for sound tooth structure, 8–15 for moderate bacterial penetration and > 15 for deep bacterial penetration may be appropriate, based on the work of Gostemeyer et al. in 2022 [18]. Such readings could inform decisions around whether further applications of AgF used alone or with KI or SnF_2_, or SDF used alone or with KI, should be undertaken, as well as at what stage a surgical intervention with placement of restoration is required. Future studies may inform such treatment decision making pathways.

As caries progresses into dentine, a variety of pigments may be produced by microorganisms [16]. High levels of pigment production may indicate a different metabolic state, compared to that geared toward proteolysis of the dentine and acid production. Changes in the type and intensity of colour changes over time, such as darkening, may suggest that the rate of lesion progression is slowing [11]. It is important to bear in mind that changes such as darkening will also affect assessment using laser fluorescence, since penetration into tooth structure will be reduced, making the measurements gained more a reflection of the most superficial parts of the dentine where light transmission is occurring. Adding to this, it is possible that treatments which cause teeth to darken, such as those based on ionic silver, could also affect light penetration into tooth structure. This is why it is best to use clinical measures, such as hardness on probing, in combination with fluorescence. In fact, previous studies have indicated that laser fluorescence measurements and visual tactile examinations when used together can result in better diagnostic outcomes for the detection of carious lesions [18, 31], so the same concept should be extended to monitoring lesions over time.

The present results regarding plaque composition indicate that, within the limitations of the present study, there is little effect of either AgF treatment on plaque area and metabolism at the time of the 3-month review. This is not unexpected, since the silver ions that are applied topically will penetrate into dentine, where they preserve the dentine collagen, and protect it from further degradation. They are not concentrated at the surface where they could alter the behaviour of dental plaque. Any silver on the surface is likely to be in the form of silver phosphate, which provides enhanced resistance to further caries, and inhibits the further progression of demineralisation [32].

In the present study, there were multiple similarities between the two AgF treatment types, despite one having KI and the other having SnF_2_, both of which would be expected to exert antimicrobial actions directly or indirectly. Stannous fluoride inhibits metabolic functions of bacteria [33, 34] while potassium iodide is considered non-toxic to bacteria (at concentration levels up to 1.6 M), and only exerts antibacterial actions due to osmotic stress when applied in a saturated solution and left in contact for 30 min [35]. However, when KI is present, the iodide ions can lead to the generation of microbicidal molecular iodine, and potentiate killing from endogenous porphyrins that act as photosensitizers. The application of light could be an important way to drive the formation of peroxyiodide, molecular iodine, extracellular free iodine (I_2_/I_3_^−^), and reactive iodine radicals [36]. There is growing evidence that using a dental curing light could enhance caries arrest by SDF [27, 37]. There is even a suggestion that variations in SDF effectiveness between anterior and posterior teeth could reflect not just how easily the treated sites can be cleaned, but also how much natural and artificial light they are exposed to [38]. Greater silver precipitation in dentine occurs when a dentine carious lesion is treated with SDF followed by a dental curing light [37]. Whether the same benefit can be gained using a curing light after applying AgF/KI or AgF/SnF_2_ needs to be investigated, since this measure would be simple to include as an additional step.

## Conclusion

This split mouth study demonstrates the effectiveness of a single application of AgF/KI or AgF/SnF_2_ in achieving arrest of active caries with high predictability (over 90%) in adult patients with special needs. The findings also show the value of a combined assessment approach using clinical visual-tactile examination combined with DIAGNOdent readings. Future studies should compare these AgF modalities with SDF and explore factors such as application time and the need for repeated applications.
